# Massive MIMO Indoor Transmissions at 38 and 65 GHz Applying Novel HBF Techniques for 5G

**DOI:** 10.3390/s22103716

**Published:** 2022-05-13

**Authors:** Concepción Sanchis-Borrás, Maria-Teresa Martinez-Ingles, Jose-Maria Molina-Garcia-Pardo

**Affiliations:** 1 Department of Technical Sciences, Universidad Católica San Antonio of Murcia, 30107 Murcia, Spain; 2 Department of Engineering and Applied Techniques, Centro Universitario de la Defensa, San Javier Air Force Base, Ministerio de Defensa-Universidad Politécnica de Cartagena, 30720 Santiago de la Ribera, Spain; mteresa.martinez@cud.upct.es; 3 Information Technologies and Communications Department, Universidad Politécnica de Cartagena, 30202 Cartagena, Spain; josemaria.molina@upct.es

**Keywords:** massive MIMO, mmWave, 38 GHz, 65 GHz, indoor communication, 5G, SFBC, hybrid beamforming

## Abstract

The 5G Infrastructure Public Private Partnership (5GPPP) has recently published a white paper about 5G service indoors, since up to now, it had mainly focused on the outdoors. In an indoor environment, the requirements are different since the propagation mechanism differs from other scenarios. Furthermore, previous works have shown that space frequency block code (SFBC) techniques applied to multiple antennas improve performance compared to single-input single-output (SISO) systems. This paper presents an experimental study in an indoor environment regarding the performance of a massive multiple-input multiple-output (mMIMO) millimeter-wave (mmWave) system based on the 5G New Radio (NR) standard in two frequency bands. In a first step, the 38 and 65 GHz bands are compared by applying a low-complexity hybrid beamforming (HBF) algorithm. In a second step, the throughput and the maximum achievable distance are studied using a new algorithm that combines the SFBC technique and HBF. Results show, at 38 GHz with HBF and aggregated bandwidths (4 × 100 MHz), a maximum throughput of 4.30 Gbit/s up to 4.1 m. At 65 GHz, the SFBC + HBF algorithm improves the communication distance by 1.34, 1.61, or 1.75 m for bandwidths of 100, 200, or 400 MHz, respectively.

## 1. Introduction

Millimeter-wave (mmWave) frequencies for 5G and 6G have been adopted as the main resource for obtaining wireless data transmission beyond Gbps [1]. The World Radio Conference 19 (WRC-19) [2] established 5G frequency bands for mmWave transmissions at 26 GHz (n258), 28 GHz (n257–261), 39 GHz (n260), 41 GHz (n259), and 47 GHz (n262). The available bandwidths were 50, 100, 200, and 400 MHz. In study [3], the 65 GHz band was presented as a band for the next generation of mobile standards.

Many measurement campaigns have been carried out to analyze the channel characteristics in the mmWave bands for both indoor and outdoor environments; see [3] (Table II) for a summary. The 5G Infrastructure Public Private Partnership (5GPPP) has published a white paper about the delivery of 5G services indoors [4]. It notes that research and innovation activities have focused primarily on outdoor use cases, as well as supporting the user and their applications while on the move. However, many use cases in indoor environments are not always adequately reflected by the eminent requirements for outdoor applications.

Massive multiple-input multiple-output (mMIMO) is necessary in mmWave systems to compensate for the path loss [5]; however, traditional beamforming systems require a dedicated radio frequency (RF) chain for each antenna, which is expensive in terms of hardware cost and power consumption [6]. Hybrid analog and digital beamforming (HBF) architecture was selected to reduce the number of RFs’ chains [7,8], and achieved high spectral efficiency. There have been many recent studies about the best HBF architectures for mmWave communications. The authors in [9] presented a manifold optimization-based algorithm, and in [10], a closed-form solution was presented that showed the optimum condition between the number of RF chains (*N_RF_*) and the number of streams (*N_S_*). Then, in study [11], this was extended to a multicarrier. Maximizing spectral efficiency using the minimum mean square error (MMSE) based on the principle of alternating optimization was presented in [12]. Furthermore, there were many other studies that ed mmWave mMIMO transmissions, presented in Table 1.

According to the authors’ best knowledge, there is a lack of research studies about experimental indoor 5G transmissions that apply HBF algorithms in mmW. In this paper, in an indoor scenario (a lab of the university), wideband channel measurements at the 38 and 65 GHz bands are used to deduce the achievable throughput when mMIMO-HBF techniques are used. Apart from 38 GHz, the 65 GHz band is also analyzed since it is considered as a feasible band for 6G [3]. In summary, the main contribution of this work is the implementation of mMIMO algorithms into real indoor measurements in two different 5G/6G frequency bands.

The algorithm described in [11] was chosen since the computational complexity is much lower than others, as claimed in [22], resulting in a spectral efficiency very similar to the algorithms defined in [9,12]. Transmission diversity is applied by combining the space frequency block code (SFBC) technique of [23,24] and the above algorithm to carry out an experimental study of the benefit of applying this new algorithm when the signal-to-noise ratio (SNR) is low.

The paper is organized as follows. In Section 2, the experimental setup and the channel sounder are described. The methodology is described in Section 3: the algorithms implemented and the physical layer parameters used. The SNR and the achievable throughput are presented and discussed in Section 4. Finally, the conclusions are presented in Section 5.

## 2. Description of the Experimental Setup

### 2.1. Scenario

**Figure 1:**
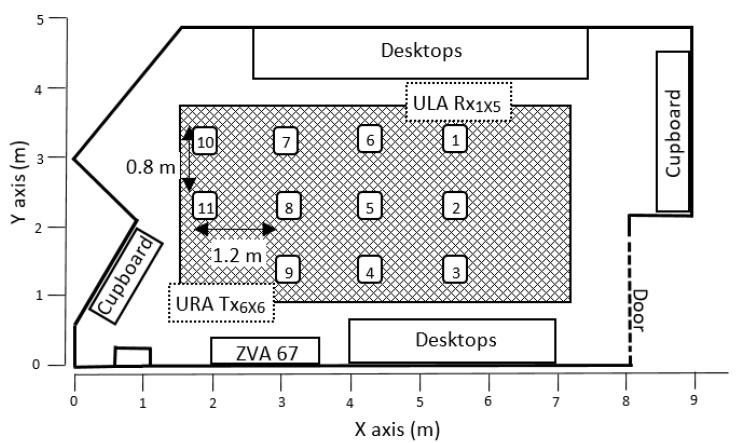
Location of Tx and Rx in the laboratory.

Measurements were carried out in a laboratory at the Universidad Politécnica de Cartagena, with a volume of 8 × 4.8 × 3.5 m^3^. Figure 1 shows a scheme of the laboratory and where the positions selected for the transmitter (Tx) and receiver (Rx) are located in it. The lab is furnished with desks, chairs, shelves, and closets, and is equipped with electronic devices such as computers.

In the measurements, eleven Rx positions have been selected to study a wide range of distances within the scenario. The distance between each Rx position and the Tx is indicated in Table 2. In order to obtain multiple antennas, the Tx antenna was located in an XY positioning system, implementing a 6 × 6 virtual uniform rectangular array (URA). Thus, 36 (6 × 6) complex channel transfer functions (CTFs) have been measured for each Tx position. For Rx, likewise, a 1 × 5 uniform linear array (ULA) was used. With this configuration, 36 × 5 mMIMO measurements were performed. The separation between antenna elements for both the URA Tx and the ULA Rx was set at 3 mm for the 38 GHz band (<λ40 GHz/2) and 2 mm for the 65 GHz band (<λ65 GHz/2). The Tx and Rx antennas were located at heights of 1.647 m and 1.544 m, respectively.

### 2.2. Channel Sounder

A Rohde & Schwarz ZVA 67 vector network analyzer (VNA) was used to measure the radio channel at both frequency bands. The system was based on measuring the transmission coefficient (*S*_21_), which measured the insertion loss in the transmission. By placing one antenna in each port, separated by a given distance, the propagation channel was measured.

**Figure 2:**
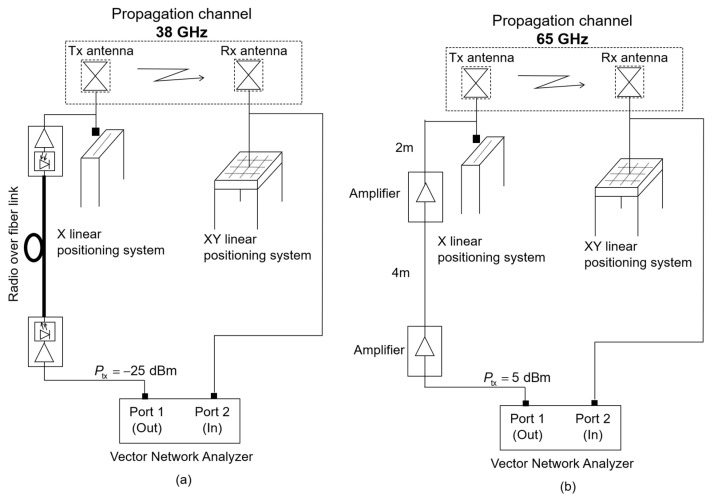
Measurement setup: (**a**) 38 GHz and (**b**) 65 GHz.

In the case of 38 GHz, a broadband radio over fiber (RoF) link converter (reference EMCORE, Optiva OTS-2, 50 MHz–40 GHz) was used to separate both antennas. In the case of 65 GHz, two coaxial cables of lengths 4 m and 2 m with insertion losses of around 5 dB/m at 62 GHz were employed. In this case, to compensate for the attenuation of the cables, two amplifiers (reference HXI HLNA-465) with a 25 dB gain were used; see Figure 2.

Electrical parameters to configure the VNA are shown in Table 3. For both bands, the system was through (THRU) calibrated to eliminate the effect of cables and amplifiers or the RoF, thus, the VNA measured the radio channel between antennas.

Regarding the antennas, at 38 GHz, two identical vertically polarized omni-directional antennas (Q-Par reference QMS-00017) were employed, i.e., one as the transmitter and one as the receiver. These antennas operate from 0.8 to 40 GHz, with typical nominal VSWR 2.5:1, a gain of −2.2 to 5.9 dB for the specific band, and 3 dB beamwidth from 18° to 160°. At 65 GHz, two identical vertical polarized antennas (Q-par QOM55-65 VRA) were used for both the transmitter and the receiver. The gain of these antennas ranged from 4.3 to 5.2 dB within the considered band, and the typical 3 dB elevation beamwidth varied from 24° to 33°, while it was omni-directional in the horizontal plane.

Finally, during the measurements, nobody was inside the environment to guarantee stationarity of the wireless channel. All the measurements were carried out in direct line-of-sight (LOS) conditions for all positions.

## 3. Methodology

The throughput was deduced with MATLAB software using the Monte Carlo method. Firstly, the physical layer was implemented according to the 5G NR Standard specifications [25]. Then, the HBF algorithms were implemented. Finally, the real channel frequency responses, obtained in the measurement campaign described in the previous section, were introduced in the software.

The following section presents the implemented algorithms (Section 3.1) and the physical layer parameters (Section 3.2) that are needed to understand the throughput analysis.

### 3.1. Implemented Algorithms

Two massive MIMO-OFDM algorithms have been simulated to study their application in indoor environments for 5G millimeter communications.

Firstly, due to its low complexity and high speed, the HBF algorithm for orthogonal frequency division multiplexing (OFDM)-based systems described in [11] was selected. Secondly, the SFBC technique [23,24] has been also added to the previous algorithm (SFBC-HBF) to experimentally investigate the benefit of applying these techniques when the SNR is low.

#### 3.1.1. Massive MIMO Hybrid Beamforming (HBF)

**Figure 3:**
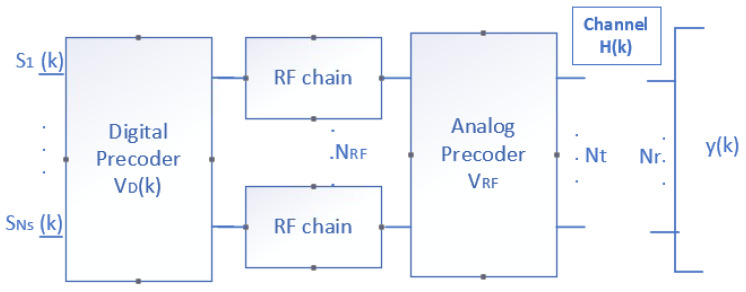
Block diagram of the HBF technique.

As shown in Figure 3, the transmitting HBF structure modified the *Ns* data streams or layers in each of the *N* subcarriers passing through the digital precoder ***V****_D_*. Then, the signals were sent over RF chains to the analog precoder $V_{RF}$, and finally, the output data were transmitted by the *Nt* antennas, and then were multiplied by the channel matrix *H.* The received signal vector $y\left(k\right)\;\in {\mathbb{C}}^{N_{r}x1}$ at the *k*th subcarrier can be expressed as:

(1) $$y\left(k\right)=H\left(k\right)V_{RF}V_{D}\left(k\right)s\left(k\right)+n\left(k\right),$$

where *k =* 1, …, *N*, $s\left(k\right)\;\in {\mathbb{C}}^{N_{s}x1}\;$, and $n\left(k\right)\;\sim \;CN\left(0,\sigma^{2}I_{Nr}\right)$ denote the transmitted symbol vector and the additive noise vector at the *k*th subcarrier, respectively. $V_{RF}$ $\in {\mathbb{C}}^{N_{t}xN_{RF}}$ and $V_{D}\left(k\right)$ $\in {\mathbb{C}}^{N_{RF}xN_{s}}$ denote the analog and digital precoder, respectively. The analog precoder was the same for all subcarriers. Finally, $H\left(k\right)\in {\mathbb{C}}^{N_{r}xN_{t}}$ was the channel matrix in the frequency domain at the *k*th subcarrier.

The $V_{RF}$ is first obtained as:

(2) $$V_{RF}\left(i,j\right)=\left\{\begin{array}{c} 1,\;\;if\;\eta_{i,j}=0,\\ \frac{\eta_{i,j}}{\left|\eta_{i,j}\right|},\;\;otherwise \end{array}\right\}$$

where

(3) $$\eta_{i,j}=\underset{l\neq i}{\sum }G_{j}\left(i,l\right)V_{RF}\left(l,j\right),$$

(4) $$G_{j}=\frac{\gamma^{2}}{\sigma^{2}}F_{1}-\frac{\gamma^{4}}{\sigma^{4}}F_{1}{\bar{V}}_{RF}^{j}C_{j}^{-1}{\left({\bar{V}}_{RF}^{j}\right)}^{H}F_{1},$$

(5) $$F_{1}=\frac{1}{N}\overset{N}{\underset{k=1}{\sum }}{\left(H\left[k\right]\right)}^{H}H\left[k\right]),$$

(6) $$C_{j}=I+\frac{\gamma^{2}}{\sigma^{2}}{\left({\bar{V}}_{RF}^{j}\right)}^{H}F_{1}{\bar{V}}_{RF}^{j}$$

and $\gamma =\sqrt{P/\left(N_{t}N_{RF}\right)},P$ being the total transmit power per subcarrier, ${\bar{V}}_{RF}^{j}$ $\mathrm{is}\;\mathrm{the}\;\mathrm{sub}-\mathrm{matrix}\;\mathrm{of}\;V_{RF}\;\mathrm{with}\;j^{\mathrm{th}}\;\mathrm{column}\;\mathrm{removed},\sigma^{2}$ is the noise power, and ${\left(\cdot \right)}^{ℋ}$ represents the conjugate transpose of (·).

For a fixed $V_{RF}$, the design of $V_{D}$ can be written as:

(7) $$V_{D}\left(k\right)=Q^{-\frac{1}{2}}U_{e}\left(k\right)\Gamma_{e}\left(k\right),$$

where $Q=V_{RF}^{H}V_{RF},U_{e}\left[k\right]$ is the set of right singular vectors corresponding to the *Ns* largest singular values of $H_{eff}\left[k\right]Q^{-1/2}$, $H_{eff}\left[k\right]=H\left[k\right]V_{RF}$ being the effective channel of subcarrier *k*. $\Gamma_{e}\left[k\right]$ is the diagonal matrix of allocated powers to each stream, i.e., assuming equal power allocation for all streams in each subcarrier, then $\Gamma_{e}\left[k\right]=\sqrt{P/N_{s}}I$.

In reception, as the number of receiving antennas was low (5 antennas), the linear decoding method was applied using Zero-Forcing (ZF) [26]. The estimation of the transmitted symbols, assuming $H_{new}\left(k\right)=H\left(k\right)V_{RF}V_{D}\left(k\right)$, is as follows:

(8) $$\overset{\wedge }{s}\left(k\right)=H_{new}{\left(k\right)}^{+}H_{new}\left(k\right)s\left(k\right)+H_{new}{\left(k\right)}^{+}n\left(k\right)$$

where ${\left(\cdot \right)}^{+}$ represents the the Penrose–Monroe pseudo inverse of (·).

#### 3.1.2. Massive MIMO Hybrid Beamforming Applying SFBC (SFBC-HBF)

The conventional SFBC-OFDM technique is a space–frequency coding; in other words, assuming *Ns* = 2, if ***s***$\;\in {\mathbb{C}}^{Nsx1}$ is the vector of input symbols whose value is [*s*1 *s*2], then the output of the SFBC mapper will be ***c***(*k*) = [*s*1 *s*2] and ***c***(*k* + 1) = [s2* − *s*1*] for each pair of subcarriers *k* and *k* + 1, respectively. Note that the spectral efficiency is the same as in the SISO case because in each carrier pair, 2 symbols were transmitted between the 2 streams. However, the spectral efficiency applying the HBF algorithm was *Ns* times higher than the SISO case, as can be seen in the results.

**Figure 4:**
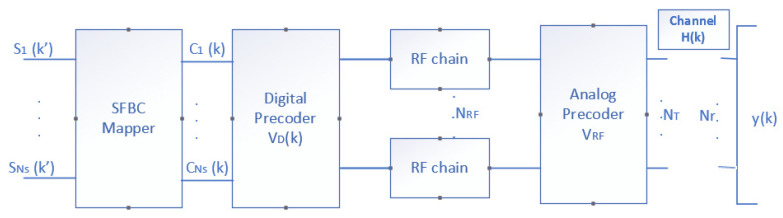
Block diagram of the SFBC-HBF technique.

Applying the SFBC technique to the input signal, the block diagram would be as follows the Figure 4:

The input signal passed through the SFBC mapper and was then sent to the $V_{D}.$ The received symbols $y\left(k\right)\;\in {\mathbb{C}}^{N_{r}x1}$ for the *k*th subcarrier can be represented by:

(9) $$y\left(k\right)=H_{new}\left(k\right)c\left(k\right)+n\left(k\right),$$

where $n\left(k\right)\;\sim \;CN\left(0,\sigma^{2}I_{Nr}\right)\;$ denotes the additive noise vector at the *k*th subcarrier.

In practice, in order to simplify the decoding and to be able to apply linear decoding, this SFBC coding was translated into the matrix by which the transmitted signal was multiplied, as was indicated using space–time coding [26]. In this case, the received signal $y\left(k^{\prime }\right)\in C^{NrNsx1}\;$ is as follows:

(10) $$y\left(k^{\prime }\right)=H_{new}^{c}\left(k^{\prime }\right)s\left(k^{\prime }\right)+n\left(k^{\prime }\right),$$

where *k*′ = 1, 2, …, *N/Ns*, and $H_{new}^{c}\left(k^{\prime }\right)\in {\mathbb{C}}^{\mathrm{NrNsxNs}}$ is the space–frequency code matrix, which, if *Ns* = 2, is defined as follows:

(11) $$H_{new}^{c}\left(k^{\prime }\right)=\left[\;\begin{array}{c} \begin{array}{cc} {h_{new}^{k^{\prime }}}_{11} & \;\;\;\;\;\;\;{h_{new}^{k^{\prime }}}_{21}\\ h_{new21}^{*(k^{\prime }+1)} & \;\;-h_{new11}^{*(k^{\prime }+1)} \end{array}\\ \vdots \\ \begin{array}{cc} {h_{new}^{k^{\prime }}}_{1N_{r}} & \;\;\;\;\;\;\;{h_{new}^{k^{\prime }}}_{2N_{r}}\\ h_{new2N_{r}}^{*(k^{\prime }+1)} & \;-h_{new1N_{r}}^{*(k^{\prime }+1)} \end{array} \end{array}\right],$$

where ${h_{new}^{k^{\prime }}}_{2N_{r}}$ is the coefficient (2, Nr) of the matrix $H_{new}\left(k\right)$ at the $k^{\prime }th$ subcarrier.

The estimation of the transmitted symbols is as follows:

(12) $$\overset{\wedge }{s}\left(k^{\prime }\right)=H_{new}^{c}{\left(k^{\prime }\right)}^{+}H_{new}^{c}\left(k^{\prime }\right)s\left(k^{\prime }\right)+H_{new}^{c}{\left(k^{\prime }\right)}^{+}n\left(k^{\prime }\right)\;$$

### 3.2. Physical Layer Parameters

The 38 and 65 GHz bands were analyzed based on the specifications for the physical layer established in the 5G New Radio (NR) standard [25]. The basic modulation scheme was OFDM, and this allowed for different subcarrier spacings [27]. A value of 120 kHz was chosen because this was an indoor environment with short distances, and very high frequencies were used. Furthermore, when the bandwidth (BW) was 400 MHz, the standard only allowed 120 kHz. From the measurements, the exact frequencies were interpolated according to the frequency band, and bandwidth were chosen to exactly fulfil the standard.

**Figure 5:**
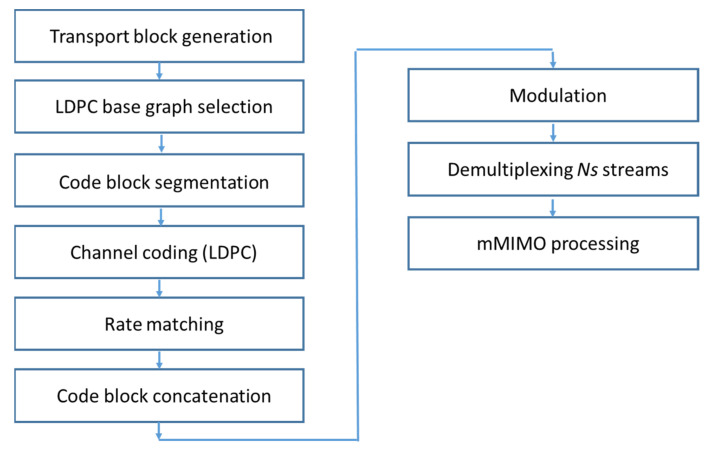
Block diagram of the physical layer.

The physical layer (PHY) was studied by focusing on the physical downlink shared channel (PDSCH). Figure 5 shows the block diagram of the physical layer. First, the transport block was generated, whose size depended on the number of subcarriers, the modulation, the *Ns*, and the code rate associated with the low-density parity check coding (LDPC) channel coder, as defined in [28]. The LDPC base graph was selected to segment the transport block into code blocks. The code blocks were then sent to an LDPC coder and to the rate matching module [29]. Next, all the code blocks were multiplexed and sent to the modulator. Finally, the modulated data were demultiplexed into *Ns* streams [30] to be processed by the algorithms described above.

The modulation and coding schemes (MCSs) chosen for the study according to the standard [28] are shown in Table 4. For each modulation, the highest coding scheme allowed by the standard was chosen in order to achieve the maximum throughput. Finally, Table 5 shows the bandwidths studied [27], with the number of resource blocks (RBs) and the number of subcarriers used in each one, since each RB has 12 subcarriers.

## 4. Results

### 4.1. SNR

**Figure 6:**
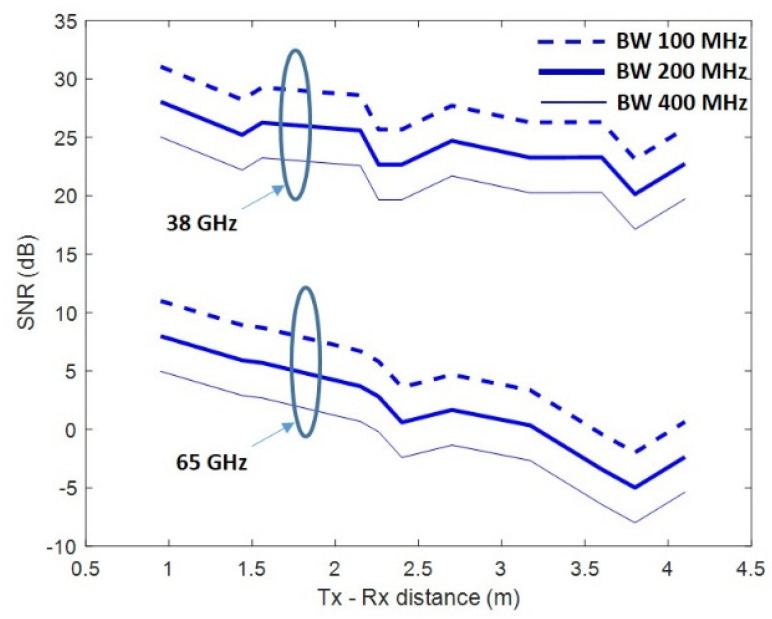
Measured SNR at 38 GHz and 65 GHz (dB).

This section presents the measured SNR for each distance and BW, where it was averaged over the number of subcarriers, shown in Table 5, and the 180 combinations (6 × 6 × 5, since a 6 × 6 URA and five antennas were used for transmission and reception, respectively). Figure 6 shows the average SNR used to obtain the throughput. It shows the SNR obtained at 38 GHz and 65 GHz in each of the studied bandwidths.

The SNR was normalized by choosing an average value of −8 dB in the worst case (65 GHz, using a bandwidth of 400 MHz, for a distance d = 3.8 m). This SNR threshold value was chosen to be able to observe variations in the throughput, since at higher SNRs, no errors occurred for the 38 GHz band. The calculation method applied was described in [31], with an equivalent isotropic radiated power (EIRP) of −9.3 dBm, and a noise power for the 100, 200, and 400 MHz bandwidths, equal to −89, −86, and −83 dBm, respectively.

The analysis showed that the SNR was higher at the 38 GHz band, and that the ratio of SNR_38GHz_/SNR_65GHz_ reached 20 dB in the best case. This value takes into account both the frequency difference and the antenna patterns. It can be observed that the SNR difference between the 100 MHz and 200 MHz bandwidths was 3 dB, while between 100 MHz and 400 MHz, it was 6 dB because of the applied noise power difference in each case. Even though SNR had been averaging, fadings were observed due to the geometry of the scenario, for example, between 2 and 2.5 m. This was due to the constructive and destructive effect of contributions from objects in the scenario.

### 4.2. Throughput Analysis

Bandwidths defined by [26] for 5G were 100, 200, and 400 MHz, which were chosen for the analysis. Simulations considered a 36 × 5 mMIMO system, and a subcarrier spacing of 120 kHz according also to the 5G recommendations. Table 4 and Table 5 show the MCSs and, for each bandwidth, the number of subcarriers chosen. In the results, the throughput for each Tx–Rx distance was calculated by applying the SNR shown in Figure 6 at each point. The parameters associated with the algorithms *Ns* and *N_RF_* were fixed at 2 and 4, respectively, assuming the condition *min*(*Nt*,*Nr*) ≥ *N_RF_* ≥ 2*Ns* for an optimum performance [10].

For statistical purposes, and in order to calculate the Packet Error Rate (PER), 100,000 transport blocks per position were first simulated, achieving a PER ≥ 10^−3^, which was low enough to allow the comparison of the different algorithms. Finally, the throughput (*Th*) was calculated from the PER as *Rb*(1-PER), where *Rb* was calculated as in [32].

#### 4.2.1. Performance According to Frequency and Bandwidth

**Figure 7:**
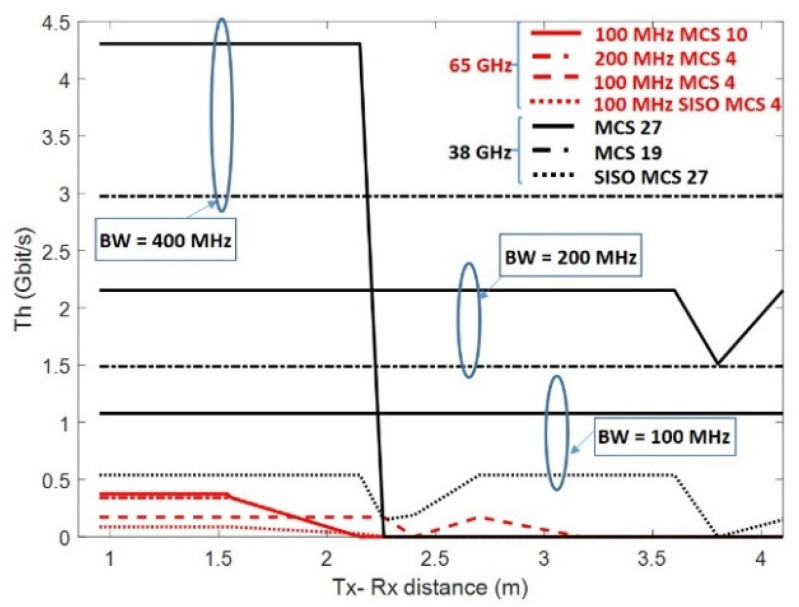
Maximum throughput at 38 and 65 GHz using HBF algorithm (BW = 100, 200, and 400 MHz).

When the HBF algorithm was used, Figure 7 shows the best throughput obtained in the 38 and 65 GHz frequency bands by applying the MCSs and BWs indicated in Table 4 and Table 5, respectively. In the 38 GHz band, only MCS 19 and MCS 27 were shown, since with MCS 4 and MCS 10, a lower throughput was obtained at all Tx–Rx distances. Applying the MSC 27 scheme and a BW of 400, 200, or 100 MHz, the maximum throughputs achieved were 4.30 Gbit/s up to 2.15 m, 2.15 Gbit/s up to 3.6 m, or 1.07 Gbit/s up to 4.1 m, respectively. Note that a higher BW implied a greater throughput, but shorter distances were achieved.

The reason for the abovementioned was that the number of subcarriers were higher, resulting in better throughput, but the SNR was lower. For example, the SNR was 6 dB lower with a BW of 400 MHz than with a BW of 100 MHz; see Figure 6. If the MSC 19 scheme was used with a BW of 400 or 200 MHz, the throughput obtained at all distances was 2.97 or 1.48 Gbit/s, respectively. At 100 MHz, the graph associated with the MSC 19 scheme was not shown, since applying MSC 27 already reached all distances with a higher throughput.

It was also observed that several aggregated bandwidths were better than a single larger one: for example, 4.30 Gbit/s was achieved up to a distance of 2.15 m when applying a BW of 400 MHz, while grouping two BWs of 200 MHz together would achieve the same throughput, but up to a distance of 3.6 m.

As shown in Figure 7, the massive MIMO HBF architecture gave better results than SISO, increasing both throughputs by a factor of 2 (*Ns*), and the Tx–Rx distance was achieved. For example, with a BW of 100 MHz and an MCS 27 scheme, a distance of 4.1 m was achieved with a throughput of 1.07 Gbit/s, while in the SISO case, 0.53 Gbit/s was achieved up to a distance of 3.6 m.

Finally, in the 65 GHz frequency band, much lower throughputs were achieved than those discussed for the 38 GHz band. This is because MCS schemes using higher order modulations (64 QAM and 256 QAM) cannot be used because the applied SNR was too low. Figure 7 shows that, up to a distance of 1.56 m, a throughput of 0.34 Gbit/s was achieved by applying a BW of 200 MHz with an MCS 4 scheme, while with a BW of 100 MHz and an MCS 4 scheme, a distance of 2.26 m was achieved with a throughput of 0.17 Gbit/s. This concludes that the SNR was a key parameter for the performance of the system.

#### 4.2.2. Performance According to Algorithm

Table 6 shows the maximum distances achieved, related to the maximum throughputs obtained for both algorithms according to the frequency band, BW, and the different MCS schemes studied. In addition, the minimum SNR required to achieve each throughput is also indicated.

At the 38 GHz frequency band, there is no improvement in throughput using the SFBC + HBF algorithm. For example, with a BW of 200 MHz and applying SFBC + HBF with the MCS 27 scheme, a throughput of 1.07 Gbit/s was achieved for all distances, while using the HBF algorithm and the MCS 19 scheme (lower modulation order), a higher throughput equal to 1.48 Gbit/s was achieved for all distances.

Nonetheless, at the 65 GHz band, performance improvements can be achieved by applying SFBC techniques because the applied SNR was much lower (see Figure 6), which means that the HBF algorithm obtains worse results than the SFBC + HBF algorithm in situations of restricted SNR. From Table 6, for a BW of 100 MHz, the HBF algorithm was best up to 1.56 m, achieving a throughput of 0.37 Gbit/s by using the MCS 10 scheme and a minimum SNR of 8.6 dB. Between 1.56 and 2.26 m, it was better to use the SFBC + HBF algorithm, with a throughput of 0.18 Gbit/s, when the same scheme and a minimum SNR of 5 dB were applied. Finally, between 2.26 and 3.60 m, a maximum throughput of 0.08 Gbit/s was achieved by using the SFBC + HBF algorithm, along with using the MCS 4 scheme and a minimum SNR of −1.3 dB. However, using the HBF algorithm, the distance reached was only up to 2.26 m, with a throughput of 0.17 Gbit/s and a minimum SNR of 3.9 dB required.

Therefore, by using the HBF algorithm, the throughput was increased by a factor of 2 (*Ns*), compared to the SFBC + HBF case if the applied SNR exceeded the minimum SNR required. However, as SFBC + HBF needed lower SNRs, the Tx–Rx distance was increased by 1.34, 1.61, or 1.75 m when a BW of 100, 200, or 400 MHz, respectively, was used. For example, for a BW of 400 MHz, and using an MCS 4 scheme, the SFBC + HBF algorithm achieved up to 2.70 m by applying a minimum SNR that was 6.6 dB lower than the one needed by the HBF algorithm, to reach 0.95 m.

In study [20], which applied a 16 QAM modulation, a BW of 800 MHz, and an EIRP = 53 dBm, a throughput of 1.2 Gbit/s was achieved to 3.5 m. However, in our case, at the 38 GHz band, when the HBF algorithm was applied, along with the MCS 10 (16 QAM) scheme, an EIRP = −9.3 dBm, and a BW of 800 MHz (8 × 100 MHz), the throughput achieved at 4.1 m was 1.4 Gbit/s.

In study [21], 16 spatial-multiplexed streams, 256 BS antennas, adaptive modulation and coding (<256 QAM), and a bandwidth 500 MHz (100 MHz × 5) were used. With these conditions, 25 Gbit/s was achieved to 10 m. However, in our case, at the 38 GHz band, when the HBF algorithm was applied, along with the MCS 27 (256 QAM) scheme, two streams, and the same bandwidth, it was possible to reach 5.35 Gbit/s at up to 4.10 m. Therefore, if 16 streams were used instead of 2, a throughput of 42.8 Gbit/s at 4.10 m was achieved. Note that our maximum distance was 4.10 m, and since our parameters were different, it was not directly comparable.

## 5. Conclusions and Future Work

In this work, massive MIMO transmissions for 5G were studied using experimental measurements in an indoor environment at 38 GHz and 65 GHz. The performance of the HBF and SFBC + HBF techniques were compared in terms of throughput, assuming the same transmitting power. The ratio of SNR_38GHz_/SNR_65GHz_ reached 20 dB in the best case. Therefore, at 38 GHz, with the HBF algorithm and with a BW of 400 MHz, a maximum throughput of 4.30 Gbit/s up to 2.15 m was achieved, while at 65 GHz, the maximum throughput was 0.68 Gbit/s up to 0.95 m.

It is better to work with aggregated bandwidths than with a single larger one. Results show that at 38 GHz, when the HBF algorithm was applied, along with aggregated bandwidths (4 × 100 MHz), a maximum throughput of 4.30 Gbit/s up to 4.1 m was achieved. However, with a single bandwidth of 400 MHz, 4.30 Gbit/s was achieved at up to 2.15 m. At 38 GHz, there was no benefit in using the SFBC + HBF algorithm, but at 65 GHz, as the applied SNR was very low, this was a reasonable solution and it improved the communication distance.

Future work will include increasing the number of antenna elements, which will allow increasing the number of streams and, therefore, the throughput. In addition, multi-user simulations, and other scenarios and algorithms, will be included in future work.

## Figures and Tables

**Table 1:** Studies that discuss mmWave mMIMO transmissions.

Reference	Year	Measurements?	Environment	Summary
[13]	2022	yes	Outdoor	It shows experimental results at 28 GHz of 256 × 16 mMIMO BS cooperation technologies for mmWave 5GE.
[14]	2020	no	-	It joins user scheduling and HBF for mmWave massive MIMO.
[15]	2020	no	-	It joins multicast beamforming and antenna selection for mmWave Massive MIMO.
[16]	2017	no	-	It presents beam division multiple access (BDMA) with per-beam synchronization (PBS) in time and frequency for mmWave/THz Massive MIMO.
[17]	2019	no	Outdoor	It applies the SD algorithm to outdoor uniform planar arrays’ (UPAs) hybrid beamforming mmW massive MIMO systems. It outperforms significantly the ZF and MMSE detectors with 16 QAM modulation over the whole range of SNR.
[18]	2017	no	Outdoor	It provides an overview of the existing multibeam antenna technologies which include the passive multibeam antennas (MBAs) based on quasi-optical components and beamforming circuits, and multibeam phased-array antennas enabled by various phase-shifting methods.
[19]	2019	no	-	It provides a hybrid precoding design for mmWave massive MIMO systems. It uses a phase pursuit technique.
[20]	2015	yes	Indoor	It presents an experimental study of 28 GHz band with 800 MHz bandwidth and beamforming based on Massive MIMO; 96 × 8 mMIMO; 16 QAM; EIRP = 53 dBm; 1.2 Gbit/s can be achieved to 3.5 m.
[21]	2019	yes	Indoor	It presents an experimental study of 28 GHz frequency band with 500 MHz (100 MHz × 5) bandwidth with 16 spatial-multiplexed streams. Number of BS antennas = 256. Adaptive modulation and coding (<256 QAM). EIRP is not indicated; 25 Gbit/s can be achieved to 10 m.

**Table 2:** Distances between transmitting and receiving antennas for each Rx position.

Rx Position	9	11	8	4	10	7	5	6	3	2	1
Tx–Rx Distance (m)	0.95	1.44	1.56	2.15	2.26	2.40	2.70	3.17	3.60	3.80	4.10

**Table 3:** Parameters for channel sounding for each band.

	38 GHz	65 GHz
VNA output power (dBm)	−25	5
Intermediate frequency filter (Hz)	100	10
Number of points	8192	2048
Measured frequency band (GHz)	1–40	57–66
Strategy to separate Tx and Rx antennas	Pre and post amplified EMCORE opto-converters	Cable and two 25 dB amplifiers

**Table 4:** Modulation and coding schemes (MCS).

MCS	Modulation	Codification (Coding Rate × 1024)
4	4 QAM	602
10	16 QAM	658
19	64 QAM	873
27	256 QAM	948

**Table 5:** Bandwidths, RBs, and number of subcarriers.

BW (MHz)	RBs	Number of Subcarriers (*N*) (RBs × 12)
100	66	792
200	132	1584
400	264	3168

**Table 6:** Comparison between HBF and SFBC + HBF.

Band (GHz)	MCS/Algorithm	BW (MHz)	Th (Gbit/s)	Max. Distance (m)	Min. SNR (dB)
65	4/HBF	100	0.17	2.26	3.9
		200	0.34	1.56	3.9
		400	0.68	0.95	3.9
	4/SFBC + HBF	100	0.08	3.60	−1.3
		200	0.17	3.17	−2.2
		400	0.34	2.70	−2.7
	10/HBF	100	0.37	1.56	8.6
		200	0	-	-
		400	0	-	-
	10/SFBC + HBF	100	0.18	2.26	5.0
		200	0.37	1.56	5.8
		400	0.22	0.95	4.9
38	19/HBF	100	0.74	4.10	14.9
		200	1.48	4.10	15.3
		400	2.97	4.10	15.4
	19/SFBC + HBF	100	0.37	4.10	9.3
		200	0.74	4.10	9.8
		400	1.48	4.10	9.7
	27/HBF	100	1.07	4.10	21.9
		200	2.15	3.60	21.2
		400	4.30	2.15	21.4
	27/SFBC + HBF	100	0.53	4.10	14.7
		200	1.07	4.10	14.9
		400	2.15	4.10	14.9
